# The National Health

**Published:** 1923-10

**Authors:** 


### THE NATIONAL HEALTH.

### THE COST OF PROGRESS.

THE Fourth Annual Report of the Ministry of Health for 1922–23 has just been issued (Stationery Office, 4s. net). It is, as usual, a very full and detailed account of the position of the public health and the progress and cost of local government.

### A Vast Expenditure.

Some conception of the vastness of the machinery of local government in England and Wales may be gained by turning to Appendix X. of the Ministry's Report. The last figures tabulated in detail are for 1920–21. They show a net expenditure out of rates of 161 millions, after crediting receipts from trading concerns, etc., and an expenditure out of Exchequer grants of 60½ millions. This is apart from capital expenditure—the loans expended during the year amounted to 94½ millions; and the debt outstanding in March, 1921, was 657 millions. In this amount is included 93 millions for Housing and Town Planning, an amount which we read elsewhere in the Report has grown to 175 millions. These enormous sums do not include expenditure on National Health Insurance, to which we refer later, but embrace such services as Education, Highways and Police; the Health services alone account for a very considerable sum and it is in regard to these that the Minister is primarily concerned in view of the concurrent Exchequer liabilities for so many of the services. The Report states:—

The provision made by Parliament for health services was less in 1922–23 than in 1921–22, and the amounts included in the Estimates for 1923–24 show a further reduction in respect of most services. The savings already secured are consistent with the maintenance of the standard of each service and with some measure of development in a number of directions; and the Estimates for next year are framed upon a basis which should allow the same policy to be followed. The greater part of the fall in cost is attributable to downward movements in prices and rates of interest.

### Co-operative Economies.

Economies in another sense have been made by the closer examination of expenses in institutions of comparable kinds. It would have been impossible, says the Report, for the Department to bring about the extension of this valuable practice without the complete assent and practical co-operation of local authorities, which has been accorded in full measure. This question of economy looms large in various sections of the Report. Thus, in regard to tuberculosis, the new policy of rationing the local authorities with grants in aid of their schemes is referred to:—

The maximum amount of expenditure which it was possible to approve for grant was not such as to permit of any important extensions of Local Authorities' schemes, other than those in respect of which contractual obligations had already been incurred. But in each case the amount approved was sufficient to enable at least the same amount of treatment to be provided as in the previous year.

Notwithstanding this, the number of beds in residential institutions approved during the year increased by 542, and it is anticipated that 1,000 additional beds will be completed during 1923–24, under the curtailed programme.

### Maternity and Child Welfare.

In the latter part of the year 1922 a local investigation of the grant-earning expenditure of some of the large local authorities was instituted in order to arrive at a standard rate of expenditure for which each of the services can be carried on efficiently and economically:—

The investigation is still proceeding, but its present results indicate that, while Local Authorities for the most part get good value for their expenditure on these services, there are some directions in which they could effect savings without impairing efficiency. Those Local Authorities, for instance, which have instituted a centralised system of purchasing supplies obtain better prices generally under their contracts than those which leave each committee to purchase supplies separately for its own needs. There is reason to believe that economies might be effected by greater attention to the working of lighting, heating and laundry plants in large institutions.

### Saving of Child Life.

In regard to midwives, it is of interest to read that the services of a trained midwife are now available for some 73 per cent. of the rural population of England. This fact has obviously had some influence upon one of the most striking phenomena of the present century—the rapid reduction of the infant mortality rate:—

At the end of the last century the average death-rate of children under one year of age in England and Wales was over 150 per thousand births; in the first ten years of the present century the rate was reduced to 128; in the next five years to 110; in 1916–20 to 90; in 1921 to 83; and in 1922 to 77 (provisional figure). It would, of course, be going too far to claim for the work which is included under the heading of Maternity and Child Welfare sole credit for this satisfactory result, but it may at least be assumed that such work has played an important part in the improvement.

### National Health Insurance.

The Report contains interesting sections on Medical Benefit, the severance of the Irish Free State Insurance system, the Societies' Schemes of Additional Benefits and the coming second valuation, as well as the special arrangements for benefits during the period of abnormal unemployment. The expenditure on benefits in England reaches 20½ millions a year, and on administration, 4 millions. In Wales the figures are 1½ millions and £300,000, respectively.

### Wasteful Expenditure on Pauperism.

With Poplar and other examples doubtless in mind, the Ministry say:—

The events of the year furnish no ground for a revision of the view expressed in last year's Report that the burden of pauperism, which, it must be remembered, falls directly or indirectly with principal weight upon the poorer wage-earners, is capable of considerable modification as a result of the administrative methods adopted, and that a substantial reduction could be secured, without causing any general hardship or distress, where it is possible to apply strict principles of administration.

### "Fine Miscellaneous Feeding."

The Report itself must be read on many interesting subjects, so varied as Regional and Town Planning (it is said that considerable progress is being made); the Medical Inspection of Aliens; new schemes of

Sewage Disposal; the Rockefeller Foundation Gift for a School of Hygiene; the formation of a National Water Policy which is in progress; and many of the other miscellaneous topics which come within the Minister's interesting survey.

### Infectious Disease.

The outstanding features for the year 1922 were the fall in the incidence of scarlet fever and diphtheria, and the increase in the number of cases of small-pox:—

108,242 cases of scarlet fever were notified during 1922, as compared with 137,073 in 1921 and 119,490 in 1920. The mortality continued to be low, being 12.7 deaths per 1,000 of notified cases. A similar decline is shown in the number of cases of diphtheria, namely, 52,153 cases in 1922 as compared with 66,506 in 1921. The mortality rate was 78 deaths per 1,000 of notified cases.

### Voluntary Hospitals.

The Voluntary Hospitals Commission have disbursed £421,000 out of the Exchequer grant of £500,000 entrusted to them for distribution:—

On a general review of the financial position of the Voluntary Hospitals they find that the deficits on hospitals accounts have been less in amount than was expected, and that the income for maintenance of existing beds has been satisfactory, new and regular sources of revenue having been opened up in many areas; but that an urgent need remains for capital expenditure to provide additional beds which would enable the present waiting lists of patients to be reduced.

### THE HEALTH OF SCOTLAND.

The Fourth Annual Report of the Scottish Board of Health has also been issued (Stationery Office, Edinburgh, 5s. net). In the main it covers similar ground to that of the English Report. With regard to the general need for economy, the Board point out that their policy has been to sanction no developments that were not urgently necessary, and to restrict existing services wherever restriction was possible without danger to the public health. The ban on developments has meant more than appears on the surface, for several of the health services—notably the school health service and maternity and child welfare—were practically new services that were only in the course of development when the need for economy arrested them.

### An Unfortunate Result.

One unfortunate result of the policy of economy is frankly stated:—

The offer of the Carnegie United Kingdom Trustees to provide a National Institute of Maternity and Child Welfare at a cost of not more than £30,000 was made under a time limit which expired during the year. As there was no possibility, in present circumstances, of making arrangements to meet the annual finance of the Institute when established, the proposal had to be allowed to lapse, and the Trustees have now withdrawn the offer.

### A Heavy Infant Mortality.

They can ill afford to refuse such offers in Scotland, where the figure of Infant Mortality (101.4) compares very unfavourably with that quoted above (77) for England. The Report has something to say about the effect of "childish complaints":—

The infant mortality rate was 101.4 per 1,000 births, which was 11.1 per 1,000 higher than the rate for 1921, the lowest recorded rate for Scotland, but which was 3 less than the mean of the rates of the preceding ten years. The estimated deaths per 1,000 children over one and under five years of age are 19.9 compared with 12.4 for 1921. The immediate causes of the increase were epidemics of measles, whooping-cough and influenza in the first half of the year. Whatever the ultimate cause, the figures, after allowing for the mortality from influenza, bring out clearly the deadly nature of so-called "childish complaints."

### The Hairmyres Sanatorium Colony.

This Colony in the County of Lanark, with accommodation for 250 patients, deserves special mention amongst the provisions for dealing with tuberculosis:—

This institution has been equipped since its inception with special occupational and training facilities of the farm colony type; for instance, forestry, pig and poultry rearing and market gardening. The need has all along been recognised for a complementary scheme of workshop training for patients who are more suited by reason of their past occupation or by inclination or aptitude for indoor craft work than for an outdoor occupation. A scheme for providing technical training in various crafts which was planned some time ago has now been carried out on a somewhat modified scale from what was originally contemplated.

### The Loch Maree Poisoning.

Among the subjects that occupy the attention of the public and press at the present time, the question of the purity and cleanliness of foodstuffs takes a prominent place:—

The poisoning outbreak at Loch Maree made a marked impression on the public mind. It was followed by the discovery of arsenic in large consignments of cocoa, a striking example of the necessity for vigilance in all directions. In both these instances the manufacturers were in a large way of business, and it is to their credit that they made great sacrifices and considerable effort in order to remove the possibility of further danger to the public health.

### The Housing Question.

It is probably no exaggeration, according to the Report, to say that the present scarcity of houses in Scotland is at least 100,000. With regard to the houses erected under the Council's schemes and occupied, the Board's Inspector says:—

"While the majority of the tenants appear to be using the houses properly, I came across several who are very unsatisfactory, and are keeping the houses in a very dirty condition. Some of them had very little furniture, and evidently belonged to a class that had been unused to a house with modern conveniences. I also came across three cases of sub-letting, in one of which there was gross overcrowding."

### National Health Insurance.

The expenditure on all benefits in 1921 was £2,800,000, and on administration £587,000. Notwithstanding the bad conditions, unemployment especially, obtaining in 1922, the claims for sickness benefit have not been unduly high:—

Considering the circumstances of the time, there is ground for satisfaction that the conclusion of a year which might have been expected to prove excessively difficult finds societies on the whole, financially and administratively, in a fairly satisfactory position.

---

**The Discoverer of Insulin.**

It is stated that Dr. Banting, the discoverer of insulin, is probably the most heavily insured individual in the world. Insurance policies amounting to about £1,000,000 have been placed on his life because of his value to science.

